# Supplementary figures and images for: Effects of non-pharmacological interventions on patients with sarcopenic obesity: A meta-analysis
Source: PLoS One. 2023 Aug 11;18(8):e0290085. doi: 10.1371/journal.pone.0290085 (PMC10420348; doi:10.1371/journal.pone.0290085)

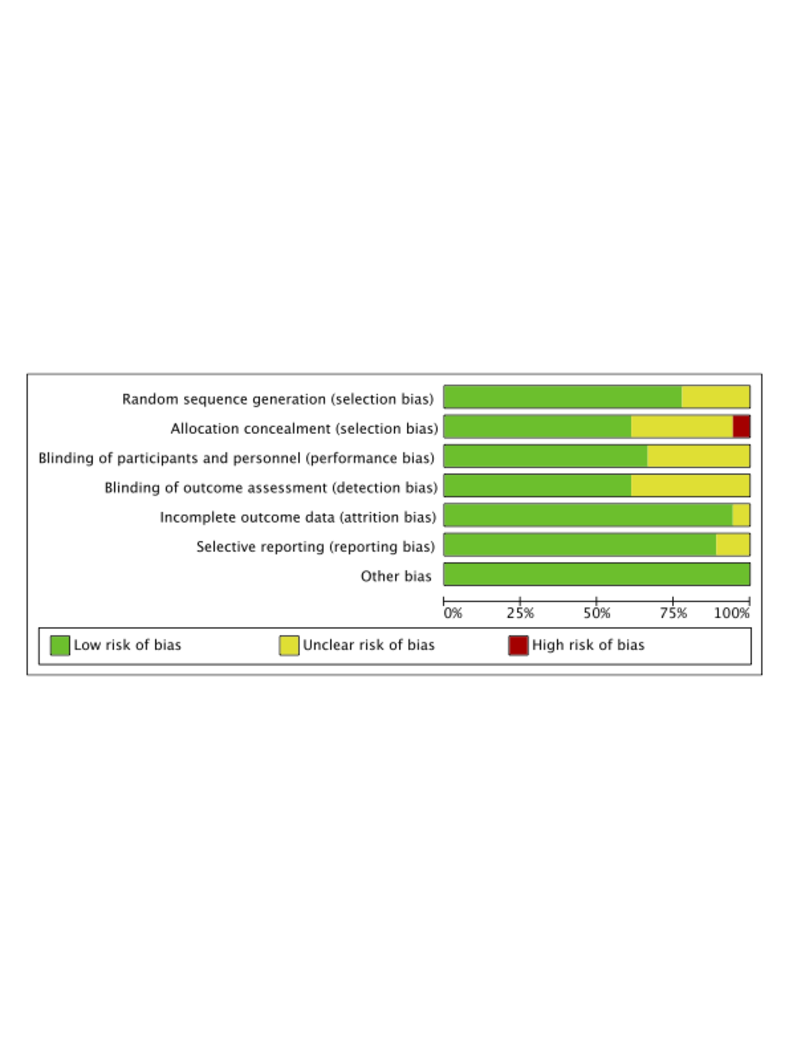

Supplement: S1 Fig — (TIF) [file pone.0290085.s002.tif]

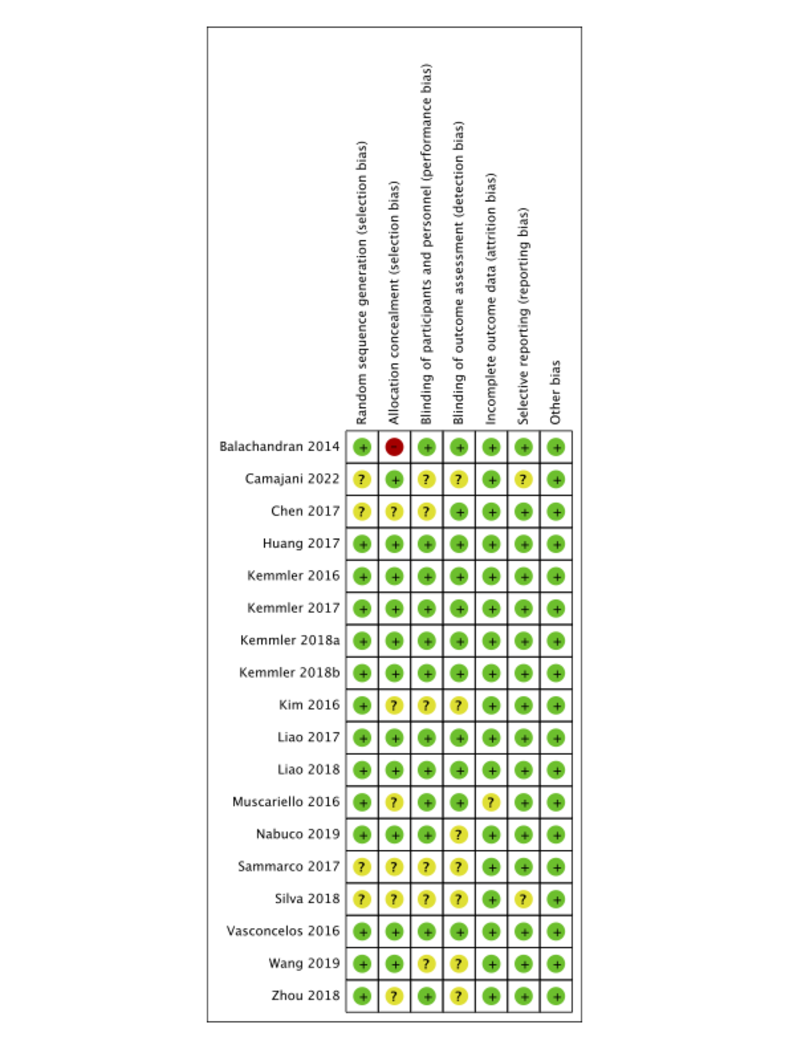

Supplement: S2 Fig — (TIF) [file pone.0290085.s003.tif]
