# Supplementary material for: Effects of non-pharmacological interventions on patients with sarcopenic obesity: A meta-analysis
Source: PLoS One. 2023 Aug 11;18(8):e0290085. doi: 10.1371/journal.pone.0290085 (PMC10420348; doi:10.1371/journal.pone.0290085)
Supplement: S1 File — (DOCX) [file pone.0290085.s004.docx]

| #1 "sarcopenic obese"[Title/Abstract] OR "sarcopenic obesity"[Title/Abstract] OR "sarcopenia obesity"[Title/Abstract] OR "obese sarcopenic"[Title/Abstract] OR "obese sarcopenia"[Title/Abstract] OR "obesity sarcopenia"[Title/Abstract]  #2 "exercise"[Title/Abstract] OR "exercise"[MeSH Terms] OR "training"[Title/Abstract] OR "physical"[Title/Abstract]  #3 "food"[Title/Abstract] OR "food"[MeSH Terms] OR "Diets"[Title/Abstract] OR "diet"[Title/Abstract] OR "Nutrition"[Title/Abstract] OR "diet"[MeSH Terms]  #4 #1 AND (#2 OR #3） |
| --- |

**Pubmed search strategy**
