# Supplementary material for: Effects of non-pharmacological interventions on patients with sarcopenic obesity: A meta-analysis
Source: PLoS One. 2023 Aug 11;18(8):e0290085. doi: 10.1371/journal.pone.0290085 (PMC10420348; doi:10.1371/journal.pone.0290085)
Supplement: S2 File — (DOCX) [file pone.0290085.s005.docx]

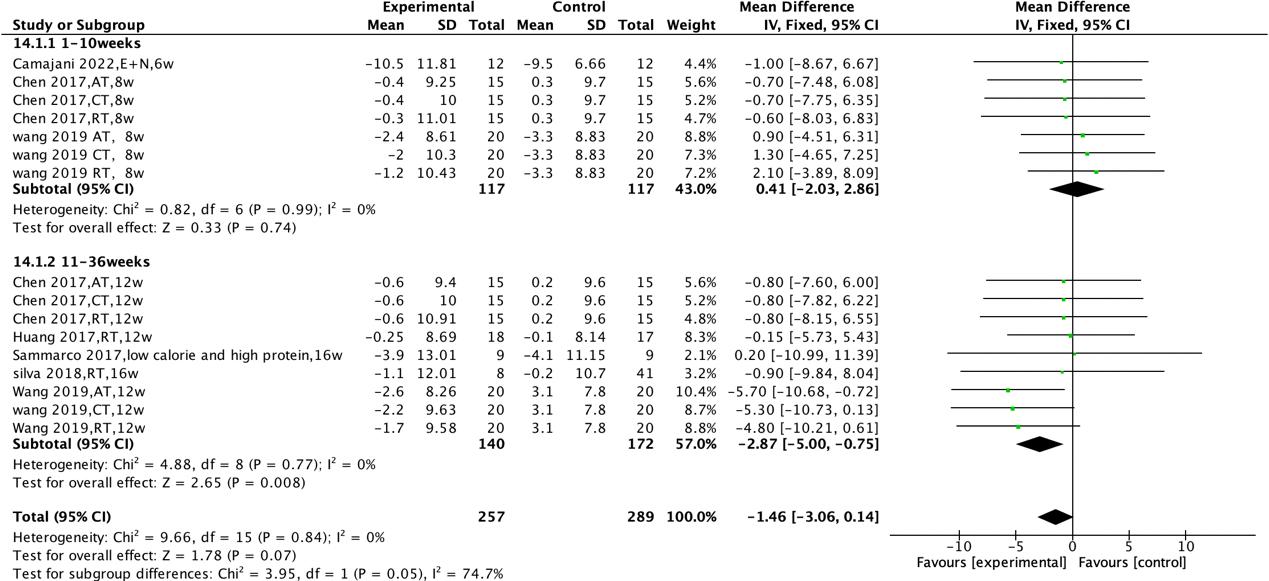


**Fig 1. Effect of time-based intervention subgroup on body weight in sarcopenic obesity patients.**


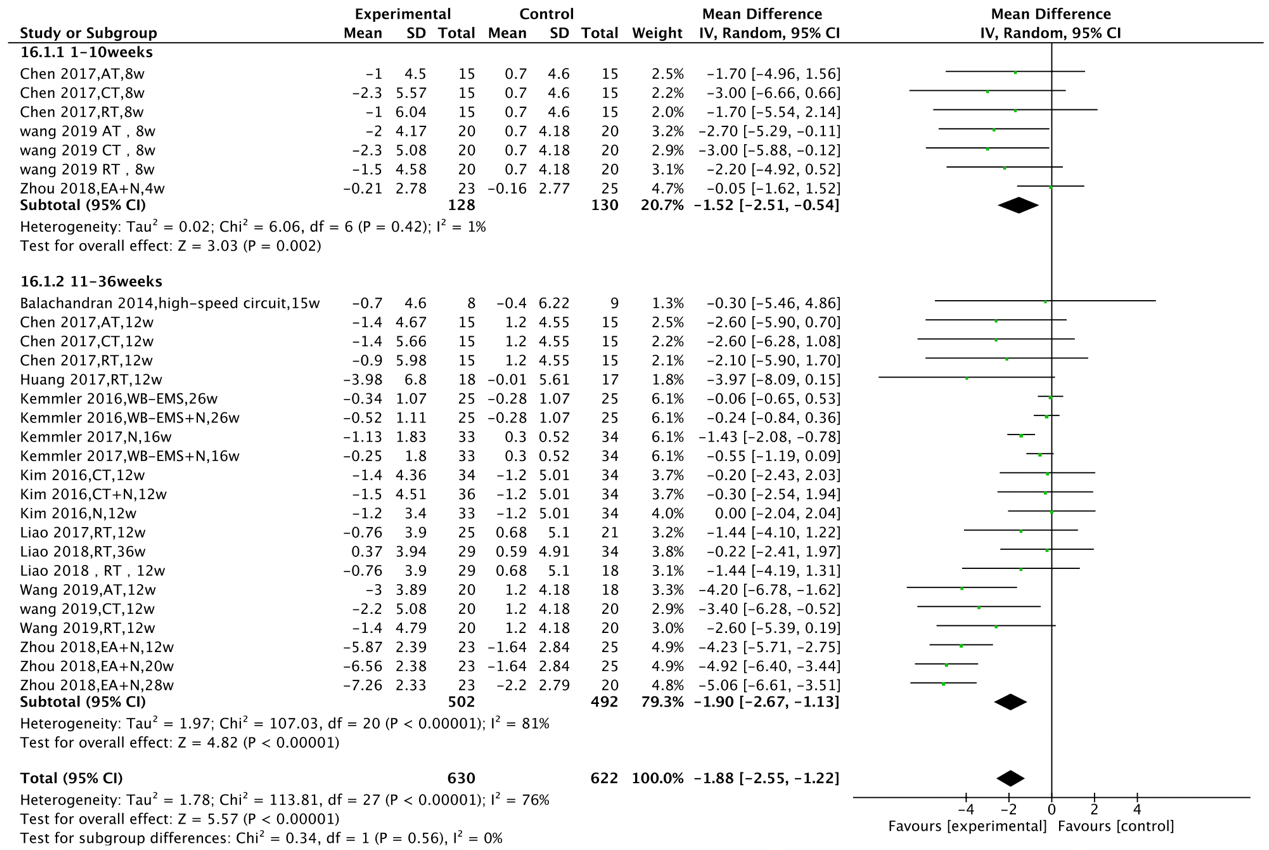


**Fig 2. Effect of time-based intervention subgroup on body fat percentage in sarcopenic obesity patients.**


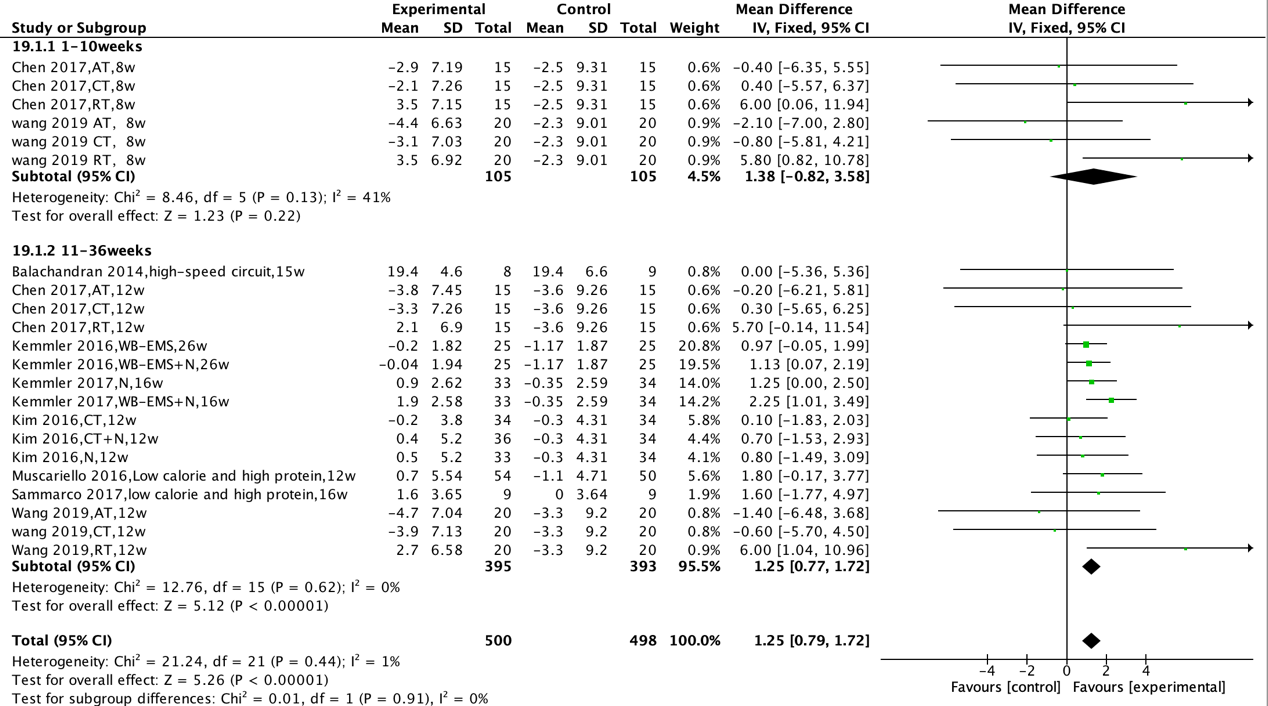


**Fig 3. Effect of time-based intervention subgroup on grip strength in sarcopenic obesity patients.**

**GS**


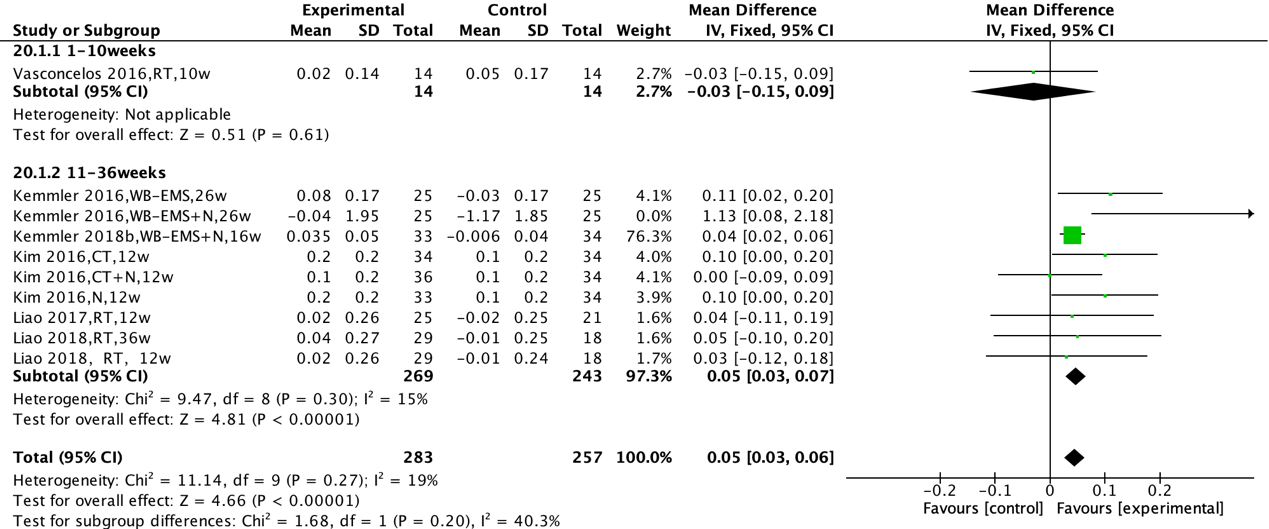


**Fig 4. Effect of time-based intervention subgroup on gait speed in sarcopenic obesity patients.**


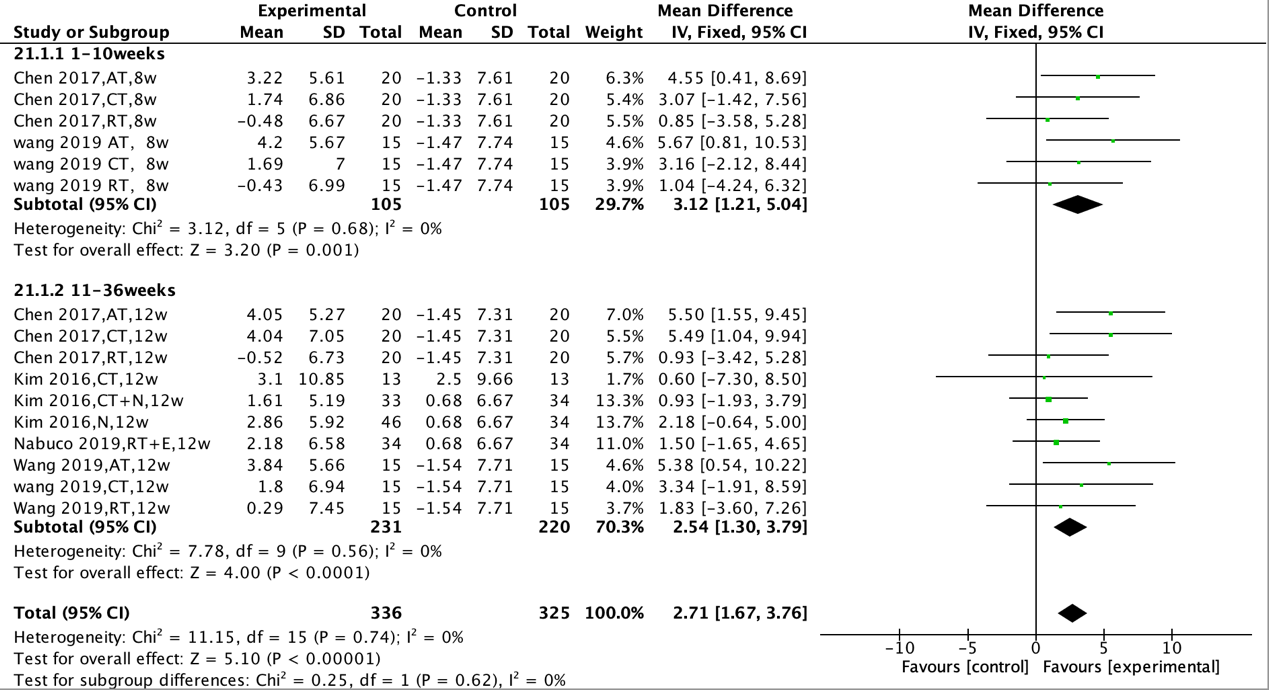


**Fig 5. Effect of time-based intervention subgroup on knee extensive strength in sarcopenic obesity patients.**
