# Supplementary material for: Effects of non-pharmacological interventions on patients with sarcopenic obesity: A meta-analysis
Source: PLoS One. 2023 Aug 11;18(8):e0290085. doi: 10.1371/journal.pone.0290085 (PMC10420348; doi:10.1371/journal.pone.0290085)
Supplement: S3 File — (DOCX) [file pone.0290085.s006.docx]

**S5 File. List of excluded 15 studies and reasons.**

| **1^st^Author** | **Year** | **Title** | **Reason for Exclusion** |
| --- | --- | --- | --- |
| **Research content was not consistent (n=7)** | | | |
| Jana Jabbour  Elisabetta Camajani  Hamed Alizadeh Pahlavani  Jinkee Park  Katharina Wittmann  Mathieu L. Maltais  Gadelha | 2022  2022  2022  2017  2016  2016  2017 | Effect of high dose vitamin D supplementation on indices of sarcopenia and obesity assessed by DXA among older adults: A randomized controlled trial  Whey Protein, L-Leucine and Vitamin D Supplementation for Preserving Lean Mass during a Low-Calorie Diet in Sarcopenic Obese Women  Exercise Therapy for People With Sarcopenic Obesity: Myokines and Adipokines as Effective Actors  Effects of 24-Week Aerobic and Resistance Training on Carotid Artery Intima-Media Thickness and Flow Velocity in Elderly Women with Sarcopenic Obesity  Impact of whole body electromyostimulation on cardiometabolic risk factors in older women with sarcopenic obesity: the randomized controlled FOrMOsA-sarcopenic obesity study  Effect of Resistance Training and Various Sources of Protein Supplementation on Body Fat Mass and Metabolic Profile in Sarcopenic Overweight Older Adult Men: A Pilot Study  Carotid Artery Parameters After Combined Exercise Training in Women with Sarcopenic Obesity | Not sarcopenic obesity patients  Lack of blank control group  Outcome indicators didn’t match  Outcome indicators didn’t match  Outcome indicators didn’t match  Outcome indicators didn’t match  Outcome indicators didn’t match |
| **Research type was not consistent (n=5)** | | | |
| Liliana Guti ́errez-L ́ opez  Yuko Gando  Doug Hershberger  Eleonora Poggiogalle  MIKYUNG RYU | 2021  2016  2015  2014  2013 | A moderate intensity exercise program improves physical function and oxidative damage in older women with and without sarcopenic obesity  Effects of resistance training on sarcopenic obesity index in older women: A randomized controlled trial  Sarcopenic Obesity: Background and Exercise Training Strategies  Treatment of body composition changes in obese and overweight older adults: insight into the phenotype of sarcopenic obesity  Association of physical activity with sarcopenia and sarcopenic obesity in community-dwelling older adults: the Fourth Korea National Health and Nutrition Examination Survey | Not RCT  It’s a manuscript  It’s a review  It’s a review  It’s a review |
| **Language was not consistent (n=1)** | | | |
| Won-Sang Jung | 2019 | Effects of 12 Weeks Combined Exercise on Physical Fitness, Isokinetic Function and IGF-1 in Elderly Women with Sarcopenic Obesity | It’s in Korean |
| **Full text was not available (n=2)** | | | |
| Valentina Muollo  Gian Pietro Emerenziani | 2021  2015 | Effects Of Strength Training Alone Or With Amino Acids In Sarcopenic Obese Adults: 747  Effects Of Aerobic Exercise Based Upon Gas Exchange Aerobic Threshold In Obese Sarcopenic Subjects. | Not found full text  Not found full text |
