# Supplementary material for: Effects of non-pharmacological interventions on patients with sarcopenic obesity: A meta-analysis
Source: PLoS One. 2023 Aug 11;18(8):e0290085. doi: 10.1371/journal.pone.0290085 (PMC10420348; doi:10.1371/journal.pone.0290085)
Supplement: Attachment — Submitted filename: Response to Reviewers.docx [file pone.0290085.s007.docx]

Review Comments to the Author

Reviewer #1:

I am honored to be invited to review this manuscript. This study conducted a meta-analysis of the effects of non-pharmacological interventions on patients with sarcopenic obesity, and the results showed that non-pharmacological interventions can effectively improve some clinical symptoms and signs of sarcopenic obesity patients, which is worth promoting and using. However, some issues should be addressed.

1.The background seems very simple. The author should provide necessary information, such as the definition scope of non-pharmacological interventions. The entire article only describes exercise therapy and nutritional support, is it comprehensive? Is there any support from the original literature?

Response:

Thanks for your kindly help, we have revised the section of Introduction according to your suggestion.

2. The inclusion criteria are inconsistent with the abstract description, should they be included in the experimental study?

Response:

It has been revised, and the experimental study is added.

3. The authors limited their search to title or abstract. For instance, “[Title/Abstract]” was used in PubMed. I am not sure that their search was comprehensive. Please explain why you limited the search to title of abstract. Moreover, other databases did not provide specific retrieval strategies.

Response:

The search strategy used a combination of subject headings and free-text terms (S1 File. Search strategy). Based on your advice, we use “All fields” to search related literature, however, the literature available for meta-analysis has not changed at all.

4. The full text number format needs to be unified, and the title should be placed below the image.

Response:

It has revised.

5. Due to different types of exercise therapy and nutritional support, the benefits for patients with different severity levels also vary, making it difficult to measure the impact of a single method. How to solve this problem?

Response:

Good question. We think this problem can be solved by using normative intervention measures, comprehensive intervention, layered intervention, individualized intervention, Long-term tracking and evaluation of the effects.

Reviewer #2:

This article has some benefits about Effects of non-pharmacological interventions on patients with sarcopenic obesity even though it has some issues which should be improved. To sum up, it is proper for scientists and student know more about this subject.

Response:

Thanks a lot for your support. We have revised it based on your suggestion.
